# The Probiotic *Bacillus subtilis* MB40 Improves Immunity in a Porcine Model of Listeriosis

**DOI:** 10.3390/microorganisms11082110

**Published:** 2023-08-18

**Authors:** Sean M. Garvey, Nima K. Emami, Justin L. Guice, Nammalwar Sriranganathan, Christopher Penet, Robert P. Rhoads, Jessica L. Spears, Rami A. Dalloul, Samer W. El-Kadi

**Affiliations:** 1 Department of Research and Development, BIO-CAT, Inc., Troy, VA 22974, USA; 2 School of Animal Sciences, Virginia Tech, Blacksburg, VA 24061, USA; 3 Department of Biomedical Sciences and Pathobiology, Virginia Tech, Blacksburg, VA 24061, USA; 4 Department of Research and Development, BIO-CAT Microbials, LLC, Shakopee, MN 55379, USA; 5 Department of Poultry Science, University of Georgia, Athens, GA 30602, USA

**Keywords:** *Bacillus subtilis*, *Listeria*, listeriosis, foodborne illness, immunity, probiotics, direct-fed microbials, swine

## Abstract

Probiotics for humans and direct-fed microbials for livestock are increasingly popular dietary ingredients for supporting immunity. The aim of this study was to determine the effects of dietary supplementation of *Bacillus subtilis* MB40 (MB40) on immunity in piglets challenged with the foodborne pathogen *Listeria monocytogenes* (LM). Three-week-old piglets (*n* = 32) were randomly assigned to four groups: (1) basal diet, (2) basal diet with LM challenge, (3) MB40-supplemented diet, and (4) MB40-supplemented diet with LM challenge. Experimental diets were provided throughout a 14-day (d) period. On d8, piglets in groups 2 and 4 were intraperitoneally inoculated with LM at 10^8^ CFU/mL per piglet. Blood samples were collected at d1, d8, and d15 for biochemical and immune response profiling. Animals were euthanized and necropsied at d15 for liver and spleen bacterial counts and intestinal morphological analysis. At d15, LM challenge was associated with increased spleen weight (*p* = 0.017), greater circulating populations of neutrophils (*p* = 0.001) and monocytes (*p* = 0.008), and reduced ileal villus height to crypt depth ratio (*p* = 0.009), compared to non-challenged controls. MB40 supplementation reduced LM bacterial counts in the liver and spleen by 67% (*p* < 0.001) and 49% (*p* < 0.001), respectively, following the LM challenge, compared to the basal diet. MB40 supplementation was also associated with decreased circulating concentrations of monocytes (*p* = 0.007). Altogether, these data suggest that MB40 supplementation is a safe and well-tolerated approach to enhance immunity during systemic *Listeria* infection.

## 1. Introduction

Probiotics are live microorganisms that confer a health benefit on a host when administered in adequate amounts [1]. Beyond supporting digestion and immunity in healthy adults [2], probiotics may also lessen the incidence or severity of certain diseases. The American Gastroenterology Association issued guidance that probiotics may be useful for (1) prevention of *Clostridioides difficile* infection in adults and children taking antibiotics, (2) prevention of necrotizing enterocolitis in preterm, low birthweight infants, and (3) management of pouchitis, a complication of inflammatory bowel disease [3]. Several meta-analyses support the use of probiotics to mitigate antibiotic-associated diarrhea [4,5] and gastrointestinal (GI) infections among infants and children [6,7]. These data highlight the potential role of probiotics in modulating the gut immune response and mitigating the expansion of pathogenic bacteria. In particular, probiotic supplementation may be an effective strategy to lessen the severity and duration of GI symptoms during foodborne bacterial infection.

Foodborne illness is one of the most prevalent GI disorders across the globe. The Centers for Disease Control and Prevention estimates 48 million cases, 128,000 hospitalizations, and 3000 deaths annually due to foodborne illness in the United States [8]. Common foodborne bacterial pathogens include *Salmonella*, *Clostridium perfringens*, *Campylobacter*, certain *Escherichia coli* strains, and *Listeria monocytogenes* (LM). LM is the Gram-positive, rod-shaped, non-spore-forming, motile, facultative anaerobe that causes listeriosis [9]. In at-risk individuals, listeriosis can lead to systemic infection, sepsis, encephalitis, meningitis, and death following the translocation of LM from intestinal tissue to mesenteric lymph nodes and visceral organs [10,11]. An estimated 1600 cases of listeriosis occur annually in the US, leading to an estimated 255 deaths and the highest rate of hospitalization among 31 known foodborne pathogens [12]. LM is genetically diverse with 14 serotypes, of which serotypes 1/2a, 1/2b, 1/2c, and 4b comprise most food and clinical isolates, and serotype 4b is most associated with human listeriosis [13,14].

Individuals can become ill with listeriosis after consuming contaminated foods such as pork, chicken, deli meats, dairy products, and vegetables [15,16]. Several post-harvest mitigation strategies are used to reduce the growth and survival of LM in food and food processing facilities (e.g., acid treatment, osmotic stress, desiccation, disinfectants, ionization radiation, and antimicrobial peptides) [17]. However, LM exhibits considerable stress tolerance and adaptability [18]. With regard to meat pre-harvest practices, antibiotics have been administered to livestock since the 1950s to improve growth performance and reduce the burden of subacute bacterial diseases that are caused by pathogens such as LM [19,20]. The emergence of antibiotic-resistant bacteria has led to policies banning the use of antibiotics in animal feed as prophylaxis and growth promoters in the European Union and the United States, respectively [21,22,23].

One alternative approach to antibiotics to improve livestock performance and increase resistance to disease is the use of beneficial, live microorganisms as feed additives, also known as direct-fed microbials (DFMs) [24,25,26]. The term DFM is often used interchangeably with probiotics. Studies dating back to the 1970s have shown a growth performance benefit of DFM supplementation in calves and piglets using strains of bacteria from the *Lactobacillaceae* family and *Bifidobacterium* genus [27,28]. Strains from the *Bacillaceae* family (including the *Bacillus* genus) have also shown benefits as DFMs [25,29,30,31]. *Bacillaceae* strains exert probiotic activity, in part, by secreting antimicrobial molecules and digestive enzymes that contribute to disease resistance and nutrient absorption, respectively [32,33,34]. *Bacillaceae*-based DFMs may also indirectly improve growth performance by promoting the growth of beneficial gut bacteria [35,36] and modulating gut and systemic immune responses [37,38,39]. *Bacillaceae* strains are particularly suited for animal DFM and human probiotic products because they can be manufactured as thermostable spores that tolerate gastric acidity following oral administration [40,41,42,43,44]. Certain probiotic strains of *Bacillus subtilis* and *Weizmannia coagulans* (formerly *B. coagulans*) have been clinically shown to support digestion and GI health in healthy human participants [45,46,47] and participants with symptoms of inflammatory bowel syndrome or dyspepsia [48,49,50,51,52,53,54,55,56].

The effects of *Bacillaceae* spore supplementation on growth performance and diarrhea severity have previously been studied in pigs [57,58,59], as well as acute challenge studies with pathogens like *C. perfringens* and *E. coli* [60,61]. However, we are not aware of any LM challenge study in pigs. *Bacillaceae* strains are especially promising antilisterial candidates, given the many reports of live *Bacillaceae* strains and cell-free supernatants with lytic or competitive growth activity toward cultured LM bacteria [62,63,64,65,66,67,68,69,70]. Therefore, we set out to study the effects of *B. subtilis* strain MB40 (MB40) as a candidate DFM in a new porcine model of listeriosis. Probiotic MB40 supplementation has previously been shown to be safe and supports GI health in clinical trials of healthy human participants [45,71]. MB40 has yet to be studied in patients with symptomatic disease or foodborne illness, so this preclinical study also served to assess the probiotic potential of MB40 to improve human immunity. We hypothesized that MB40 supplementation would improve the immune response, limit visceral dissemination of LM, and reduce intestinal histopathology following intraperitoneal LM challenge in weaned piglets.

## 2. Materials and Methods

### 2.1. Animals and Diets

This study was approved by and conducted under the guidelines of the Virginia Tech Institutional Animal Care and Use Committee (IACUC-18-010-APSC; August 2018). A total of 32 cross-bred, weaned piglets (21 days old) were acquired from the Virginia Tech swine farm and transported to a biosafety level 2 facility. The animals were individually housed and fed ad libitum a standard, non-medicated, pelleted, swine starter commercial diet (Big Spring Mill, Elliston, VA, USA) for 9 days while acclimating to the facility in environmentally controlled rooms maintained at 24 °C with a standard light cycle (12 h light/dark). The diet contained crude protein (18% minimum), crude fat (3% minimum), crude fiber (4% maximum), calcium (1% minimum, 1.5% maximum), and phosphorus (0.5% minimum). The following ingredients were listed on the label: two-grain products, processed grain by-products, plant protein products, animal protein products, forage products, cane molasses, sugar, salt, dicalcium phosphate, monocalcium phosphate, calcium carbonate, potassium chloride, iron sulfate, cobalt sulfate, copper sulfate, manganese sulfate, manganous oxide, zinc sulfate, zinc oxide, red iron oxide, calcium iodate, sodium selenite, vitamin A supplement, vitamin D3 supplement, vitamin E supplement, menadione sodium bisulfite complex (source of vitamin K activity), vitamin B-12 supplement, choline chloride, niacin, calcium pantothenate, riboflavin supplement, and mineral oil. No vaccinations were provided since the animals were in isolation in clean rooms.

**Figure 1:**
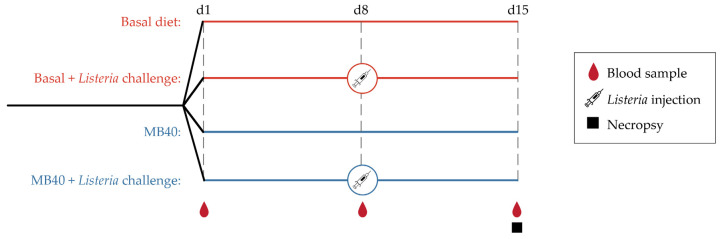
Experimental design.

Following acclimation, animals were randomly assigned to 4 experimental groups in a 2 × 2 factorial arrangement (2 dietary treatments, with and without LM challenge): (1) basal diet (Basal), (2) basal diet and LM challenge (Basal + LM), (3) MB40-supplemented diet (MB40), and (4) MB40-supplemented diet and LM challenge (MB40 + LM, Figure 1). Each experimental group included 4 gilts and 4 barrows. *Bacillus subtilis* strain MB40, registered as American Type Culture Collection (ATCC) No. PTA-122264 was derived from the *Bacillus subtilis* Marburg type strain DSM 10 (ATCC No. 6051) and displayed enhanced spore stability without genetic engineering. MB40 has also demonstrated a robust safety profile and probiotic characteristics in cell culture experiments and clinical trials [45,71]. Additionally, the United States Food and Drug Administration issued a “no objection letter” for the use of MB40 in foods according to a “generally regarded as safe” (GRAS) dossier [72]. MB40 was manufactured and provided by BIO-CAT Microbials, LLC (Shakopee, MN, USA). Briefly, MB40 was grown using aerobic fermentation methods to achieve nearly 100% spores. After fermentation, pure MB40 spore culture was concentrated via centrifugation and then spray-dried using maltodextrin as a carrier. In this animal study, MB40 was supplemented at 7.5 billion colony-forming units (CFU) per day by mixing 1 g MB40 (7.5 billion CFU/g) with the basal diet in pelleted form. Experimental diets were fed to animals for 14 days. All diets were formulated to meet or exceed the 2012 National Research Council nutrient recommendations for weaned piglets [73] and did not contain antibiotics or pharmaceutical levels of copper or zinc.

### 2.2. Bacterial Challenge

*L. monocytogenes* (4b strain) was sub-cultured from a frozen stock on blood agar plates and enriched overnight in brain heart infusion (BHI) broth. Log dilutions of BHI broth were plated on blood agar in order to determine CFU/mL. After counting and diluting to the appropriate dose, the culture was centrifuged to remove the broth, and the bacterial pellet was resuspended in phosphate-buffered saline (PBS) at 10^8^ CFU/mL before a single intraperitoneal injection at 1 mL/animal in groups 2 and 4 at d8. The non-challenged groups (1 and 3) received a similar 1 mL injection of sterile PBS. Intraperitoneal injection models the visceral spread of LM and avoids confounding variables associated with oral dosing (e.g., gastric pH, gastric volume of chyme, intestinal pH, intestinal volume of digesta). The intraperitoneal LM dose was chosen to cause systemic infection and mild GI distress without mortality. Animals were euthanized at d15, 7 days after the single dose of LM. It has previously been recognized that systemic inflammation and visceral infection can be sustained for up to 10 days following intraperitoneal bacterial pathogen challenge with LM in mice [74,75].

### 2.3. Necropsy and Sample Collection

Animals were individually weighed, and feed refusals were measured daily to determine daily feed intake throughout this study to calculate average daily weight gain (ADG), average daily feed intake (ADFI), and feed conversion ratio. Animals did not fast before sample collection. At d1, d8, and d15, blood samples were collected for standard serum chemistry and hematological profiling. Blood samples were collected in EDTA or lithium heparin BD Vacutainer^®^ blood collection tubes (Becton, Dickinson and Company, Franklin Lakes, NJ, USA) and stored on ice. Blood samples collected in EDTA-coated tubes were transported on ice for hematologic analysis. Plasma was separated from blood collected in lithium heparin-coated tubes at 4 °C for 10 min at 2500× *g* (Eppendorf, Hamburg, Germany), and aliquots were stored at −80 °C until further analysis. Fresh plasma samples were sent to the Virginia-Maryland College of Veterinary Medicine (Blacksburg, VA, USA) for blood chemistry analysis using the AU480 Chemistry Analyzer with ion-selective electrode (Beckman Coulter, Inc., Brea, CA, USA). At the end of this study (d15), animals were euthanized with an overdose (1 mL/5 kg body weight) of pentobarbital sodium (390 mg/mL) and phenytoin sodium (50 mg/mL) and exsanguinated. Abdomens were opened, and livers, kidneys, and spleens were aseptically collected and weighed. Intestinal samples were flushed with ice-cold sterile saline to remove digesta prior to weighing. Samples from the liver, spleen, and small intestine were snap-frozen in liquid nitrogen and stored at −80 °C. Fresh liver and spleen samples from animals challenged with LM were cultured for CFU enumeration.

### 2.4. Plasma Cytokine Analysis

Plasma tumor necrosis factor-alpha (TNF-α), interleukin (IL)-10, and IL-6 concentrations were determined using porcine-specific, enzyme-linked immunosorbent assay kits (Catalog Nos. EPT0015, EPI0030, and RK09048, ABclonal, Inc., Woburn, MA, USA) according to manufacturer’s instructions. Samples and standards were assayed in duplicate using a plate reader (BioTek Instruments, Inc., Winooski, VT, USA). The assays and analyses were performed according to the manufacturer’s protocol for each kit.

### 2.5. Intestinal Morphology

Following euthanasia, intestinal segments were collected, flushed with cold PBS, and fixed in 10% neutral buffered formalin. Intestinal segments were removed from the fixative and sliced into five 10 mm sections, and placed into a tissue cassette. Tissue samples were dehydrated through a graded alcohol series, cleared with xylene, and embedded in paraffin wax. Tissue samples were then sliced into 5 µm sections using a microtome, mounted onto slides (5 sections per slide), and stained with hematoxylin and eosin stain for determination of gross morphology of intestinal villus height (VH) and crypt depth (CD). Images of intestinal sections were taken using an OLYMPUS^®^ BX50 microscope (Evident Corporation, Tokyo, Japan) equipped with a Nikon Digital Sight camera. Five cross-sections per slide per animal were viewed, and a total of 12 VHs and 12 CDs were photographed for each animal, and analyzed using NIS-Elements AR 3.10 software (Nikon Instruments Inc., Melville, NY, USA).

### 2.6. Statistical Analysis

To investigate temporal effects, data were considered in repeated measures analyses. Log10 transformation was applied to the quantification of bacterial groups, and, if necessary, to some dependent variables to improve model assumptions of normality. All other data were analyzed by analysis of variance (ANOVA) using GraphPad Prism version 9.2.0 for Windows (GraphPad Software, Inc., San Diego, CA, USA). The experimental arrangement was a 3-way factorial design with two between-factors (diet and LM challenge) and one within-factor (time). The full model for the ANOVA analysis was:

*Y_nijk_* = *µ* + *α_i_* + *β_j_* + *γ_k_* + (*αβ*)*_ij_* + (*αγ*)*_ik_* + (*βγ*)*_jk_* + (*αβγ*)*_ijk_* + ε*_nijk_*

where:

*Y_nijk_*: *n*^th^ observation at level *i* of level *α*, level *j* of level *β*, level *k* of level *γ;*

*µ*: overall data mean;

*α_i_*: *i*^th^ level of the factor diet;

*β_j_*: *j*^th^ level of the factor LM challenge;

*γ_k_*: *k*^th^ level of the factor time;

(*αβ*)*_ij_*: interaction term between the diet and LM challenge factors;

(*αγ*)*_ik_*: interaction term between the diet and time factors;

(*βγ*)*_jk_*: interaction term between the LM challenge and time factors;

(*αβγ*)*_ijk_*: interaction term between the diet, LM challenge, and time factors;

*ε_nijk_*: error term.

A simplified model without time as a factor was used to evaluate differences between factors across each timepoint:

*Y_nij_* = *µ* + *α_i_* + *β_j_* + (*αβ*)*_ij_* + ε*_nij_*

Differences were considered significant if *p* < 0.05. When a significant effect was determined by ANOVA, means were compared using Fisher’s least significant differences post hoc test. *p* values for the full and simplified models for growth parameters, organ weights, hematology, plasma cytokines, intestinal morphology, and plasma biochemistry are provided as Supplementary Tables (Tables S2–S4).

Bacterial counts in the liver and spleen of LM-challenged animals were analyzed by unpaired *t*-test, and correlation analysis was used to assess the relationship between organ bacterial count and blood monocyte concentration.

## 3. Results

### 3.1. Growth Parameters

Growth performance and feed conversion ratio (FCR) were evaluated during dietary MB40 supplementation (d1–d15) and following LM challenge at d8 (see Figure 1 for schematic of experimental design). The average body weight of all animals at d1 was 7.12 ± 1.70 kg. Baseline body weights (d1) did not differ between diets (Table 1). There was a main effect of time on body weight, weight gain, average daily weight gain (ADG), and average daily food intake (ADFI), whereby animals consumed more feed and gained more weight throughout the 14 days of this study (Table 1). Neither diet (*p* = 0.672) nor LM challenge (*p* = 0.739) had significant effects on body weight. Neither feed intake nor FCR was significantly different across diets (Table 1). There was a main effect of LM challenge on FCR, whereby feed conversion was less efficient in the LM-challenged animals from d1–d15 (*p* = 0.031); however, this effect was not significant following LM challenge from d8–d15 (*p* = 0.539, Table 1). No overt symptomology of gastrointestinal illness, including diarrhea, was observed in any animals.

### 3.2. Organ Weights and Bacterial Counts

Average weights of small intestine, liver, kidney, and spleen generally increased in LM-challenged groups compared with non-challenged controls (Table 2). A main effect of diet was observed for small intestine weight, in which the MB40-supplemented animals had lower weights (*p* = 0.036); however, this effect was no longer observed following normalization to body weight (Table 2). Normalized spleen weights were significantly higher after the LM challenge (*p* = 0.017), and normalized kidney weights were slightly higher after the LM challenge (*p* = 0.052). 

**Figure 2:**
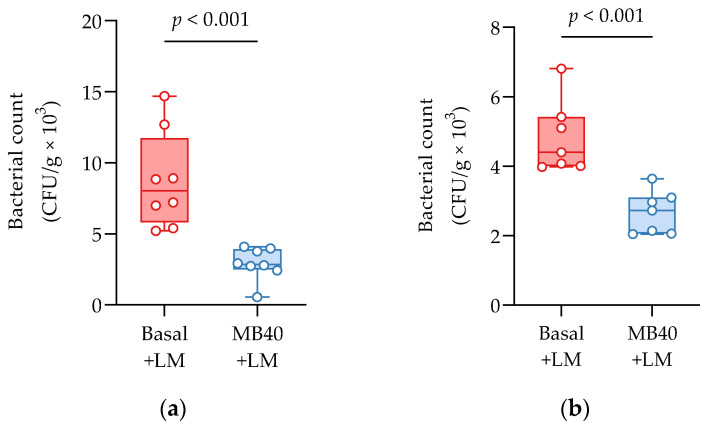
(**a**) Liver and (**b**) spleen bacterial counts 7 days following intraperitoneal *Listeria* challenge (LM) in animals fed basal diet (Basal) or diet supplemented with *B. subtilis* MB40 spores (MB40). The box and whisker plots show the median, interquartile range, and minimum and maximum values (*n* = 8 per group). Statistical differences were determined by unpaired *t*-tests.

Bacterial invasion of the liver and spleen in challenged animals was investigated by microbial enumeration. Samples from non-challenged piglets were negative for LM. MB40-supplemented animals showed a 67% reduction in liver bacterial counts compared to challenged animals fed basal diet without MB40 (2.91 ± 1.14 vs. 8.75 ± 3.38 CFU × g^−1^ × 10^3^, *p* < 0.001, Figure 2a). MB40 supplementation was also associated with a 49% reduction of bacterial counts in the spleen following LM challenge, compared to basal diet (2.46 ± 0.81 vs. 4.84 ± 0.96 CFU × g^−1^ × 10, *p* < 0.001, Figure 2b). The presence of bacteria in the liver and spleen, as well as the increased spleen and kidney organ weights, suggest that the dose of LM in this intraperitoneal injection model of listeriosis was sufficient to evoke hepatic and splenic infection and inflammation. Moreover, MB40 supplementation may have mitigated the visceral dissemination of LM from the peritoneal cavity to the liver and spleen.

### 3.3. Hematology and Plasma Cytokines

**Figure 3:**
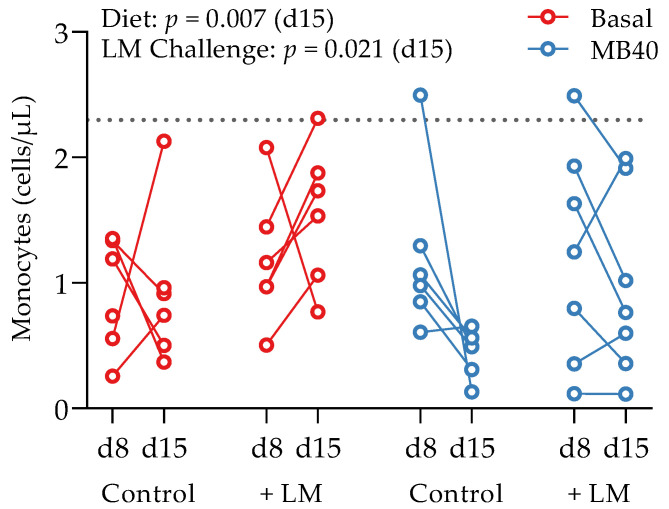
Effects of *B. subtilis* MB40 supplementation and *Listeria* challenge on blood monocyte concentrations. The line connecting data from individual animals (circles) shows the change from d8 to d15. The dotted line shows the upper reference limit (2.3 cells/µL) for pigs up to 6 weeks of age [76]. Basal, basal diet; Control, no LM challenge; d, day; LM, *Listeria monocytogenes* challenge at d8; MB40, MB40-supplemented diet.

Whole blood and plasma were collected for hematology and cytokine analysis, respectively, from animals at baseline (d1), before LM challenge (d8), and at the end of study (d15). There was a main effect of time on erythrocyte concentration (*p* = 0.039), hematocrit (*p* < 0.001), hemoglobin concentration (*p* < 0.001), packed cell volume (*p* < 0.001), and segmented neutrophil concentration (*p* = 0.020), with values lower at d15 compared to d1 (Table 3). Lymphocytes tended to increase by d8 and d15 compared to d1 (*p* = 0.087), with no effects of diet or LM challenge (Table 3). A main effect of LM challenge was significant for increased leukocyte concentrations (*p* = 0.027), including neutrophils (*p* = 0.004), and monocytes (*p* < 0.021) in LM-challenged animals at d15 compared to non-challenged animals (Table 3). Despite the inflammatory response to LM challenge and increased hepatic and splenic bacterial load, the concomitantly increased concentrations of neutrophils and monocytes fall within standard reference ranges for 6-week-old pigs [76]. A time × diet effect was significant for eosinophil concentrations (*p* = 0.032); however, elevated baseline concentrations in the animals prior to MB40 supplementation are confounding (Table 3). More telling for the impact of MB40 on potentially reducing inflammation, a main effect of diet on decreasing monocyte concentrations was observed at d15 (*p* = 0.007), whereby monocytes in MB40-supplemented animals decreased after LM challenge (d8–d15) and increased in LM-challenged animals fed the basal diet without MB40 (Figure 3).

**Table 3:** Effects of *B. subtilis* MB40 supplementation and *Listeria* challenge on hematology and plasma cytokines ^1^.

	Condition				*p* Value		
Parameters (Units)	Basal	Basal + LM	MB40	MB40 + LM	Diet	Challenge	D × C
Erythrocytes (cells/µL) *							
d1	6.7 ± 0.62	6.6 ± 0.69	6.5 ± 0.42	6.8 ± 0.39	0.926	0.593	0.331
d8	6.3 ± 0.66	6.2 ± 0.88	6.0 ± 0.78	6.5 ± 0.37	0.880	0.502	0.334
d15	6.5 ± 0.52	6.3 ± 0.68	6.3 ± 0.53	6.5 ± 0.59	0.796	0.964	0.429
Hemoglobin (g/dL) *							
d1	12.0 ± 0.80	11.9 ± 0.91	11.9 ± 0.91	12.6 ± 0.66	0.358	0.437	0.207
d8	10.9 ± 0.50	11.1 ± 1.50	10.6 ± 1.39	11.5 ± 0.38	0.847	0.191	0.415
d15	11.4 ± 0.59	11.3 ± 0.56	11.2 ± 0.92	11.3 ± 0.92	0.674	0.971	0.605
Hematocrit (%) *							
d1	39.1 ± 2.90	38.3 ± 2.56	38.9 ± 2.91	40.7 ± 2.38	0.289	0.610	0.205
d8	35.6 ± 2.11	36.0 ± 4.89	34.5 ± 4.06	37.0 ± 1.22	0.989	0.275	0.444
d15	37.2 ± 1.73	35.8 ± 3.28	35.4 ± 2.81	36.9 ± 2.62	0.721	0.985	0.176
Packed cell volume (%) *							
d1	37.1 ± 1.66	36.7 ± 2.81	37.8 ± 3.17	39.0 ± 2.22	0.132	0.658	0.376
d8	35.1 ± 3.40	35.3 ± 4.41	34.3 ± 5.16	36.0 ± 1.78	0.974	0.547	0.650
d15	36.4 ± 1.74	33.8 ± 2.93	36.0 ± 3.50	35.6 ± 2.39	0.508	0.146	0.297
Leukocytes (cells/µL) ^‡^							
d1 ^‡^	15.0 ± 1.98 ^A^	15.5 ± 3.10 ^B^	12.4 ± 2.29 ^A^	16.8 ± 3.95 ^B^	0.564	***0.047***	0.104
d8	18.4 ± 5.13	17.7 ± 6.39	15.1 ± 3.79	16.6 ± 2.68	0.233	0.835	0.561
d15 ^‡^	13.6 ± 3.35 ^A^	16.9 ± 3.03 ^B^	12.0 ± 3.22 ^A^	17.2 ± 4.93 ^B^	0.647	***0.005***	0.480
Segmented neutrophils (cells/µL) *^,‡^							
d1	7.3 ± 2.32	9.1 ± 2.54	6.6 ± 2.58	8.9 ± 3.45	0.689	0.062	0.806
d8	9.4 ± 4.20	10.4 ± 4.92	7.2 ± 3.39	9.1 ± 1.35	0.242	0.313	0.773
d15 ^‡^	4.6 ± 1.70 ^A^	8.2 ± 2.33 ^B^	5.5 ± 1.03 ^A^	7.5 ± 2.45 ^B^	0.921	***0.001***	0.285
Lymphocytes (cells/µL)							
d1	6.5 ± 1.83	5.1 ± 1.00	4.9 ± 1.35	5.0 ± 1.46	0.159	0.267	0.191
d8	7.4 ± 1.20	6.1 ± 2.46	6.3 ± 1.70	6.0 ± 1.67	0.417	0.255	0.468
d15	7.8 ± 1.93	6.7 ± 2.08	5.8 ± 3.17	7.0 ± 2.07	0.363	0.999	0.190
Eosinophils (cells/µL) **							
d1	0.17 ± 0.23	0.04 ± 0.07	0.20 ± 0.16	0.26 ± 0.20	0.072	0.645	0.152
d8 ^†^	0.21 ± 0.28 ^a^	0.07 ± 0.08 ^a^	0.17 ± 0.19 ^b^	0.25 ± 0.17 ^b^	***0.040***	0.690	0.369
d15	0.16 ± 0.09	0.29 ± 0.26	0.11 ± 0.16	0.21 ± 0.18	0.350	0.130	0.825
Monocytes (cells/µL) ^‡,^**							
d1 ^‡^	1.0 ± 0.43 ^A^	1.2 ± 0.65 ^B^	0.75 ± 0.29 ^A^	1.5 ± 0.72 ^B^	0.870	***0.047***	0.234
d8	0.91 ± 0.46	1.2 ± 0.53	1.5 ± 0.88	1.2 ± 0.86	0.316	0.928	0.377
d15 ^†,‡^	0.89 ± 0.59 ^a,A^	1.6 ± 0.68 ^b,A^	0.44 ± 0.17 ^a,B^	0.97 ± 0.73 ^b,B^	***0.007***	***0.021***	0.801
Interleukin-10 (pg/mL) *							
d1	326 ± 104.4	401 ± 153.0	354 ± 148.1	361 ± 154.5	0.903	0.425	0.500
d8	285 ± 73.6	343 ± 106.2	360 ± 66.5	317 ± 87.1	0.430	0.803	0.103
d15	283 ± 70.1	235 ± 54.4	287 ± 62.0	276 ± 54.1	0.299	0.190	0.399
TNF-α (pg/mL) *							
d1	512 ± 156.1	595 ± 243.0	435 ± 102.0	452 ± 163.5	0.092	0.434	0.605
d8	433 ± 220.8	410 ± 151.1	458 ± 177.5	330 ± 123.8	0.659	0.228	0.398
d15	275 ± 123.5	337 ± 147.0	407 ± 194.0	403 ± 207.8	0.123	0.646	0.599
Interleukin-6 (pg/mL) *							
d1	736 ± 152.8	854 ± 152.6	756 ± 300.7	775 ± 281.6	0.726	0.409	0.551
d8	336 ± 91.3	427 ± 171.7	439 ± 102.0	421 ± 137.7	0.299	0.435	0.242
d15	505 ± 70.0	474 ± 141.2	504 ± 134.5	492 ± 106.3	0.837	0.608	0.818

^1^ Data are means ± standard deviation (*n* = 6–8 per group); * main effect of time; ** time × diet interaction; ^†^ main effect of diet; ^‡^ main effect of LM challenge.; significant differences between groups (*p* < 0.05, ANOVA) are denoted by unshared letters (diet factor: a, b; challenge factor: A, B); significant *p* values are italicized and bold (*p* values for three-way repeated measures ANOVA are shown in Table S2). Abbreviations: Basal, basal diet; d, day; D × C, diet × challenge interaction; LM, *Listeria monocytogenes* challenge at d8; MB40, MB40-supplemented diet; TNF-α, tumor necrosis factor-alpha.

There was a main effect of time on plasma cytokine concentrations (*p* < 0.05, Table 3). Whereas IL-10 concentration was highest in animals at d1 and lowest at the end of study, TNF-α and IL-6 were greatest at d1, generally decreased by d8, but increased at d15 (Table 3). There were no differences in plasma cytokine concentrations across diet or LM challenge. The significant changes in hematology and plasma cytokine concentrations over time likely represent typical immune maturation in piglets.

**Figure 4:**
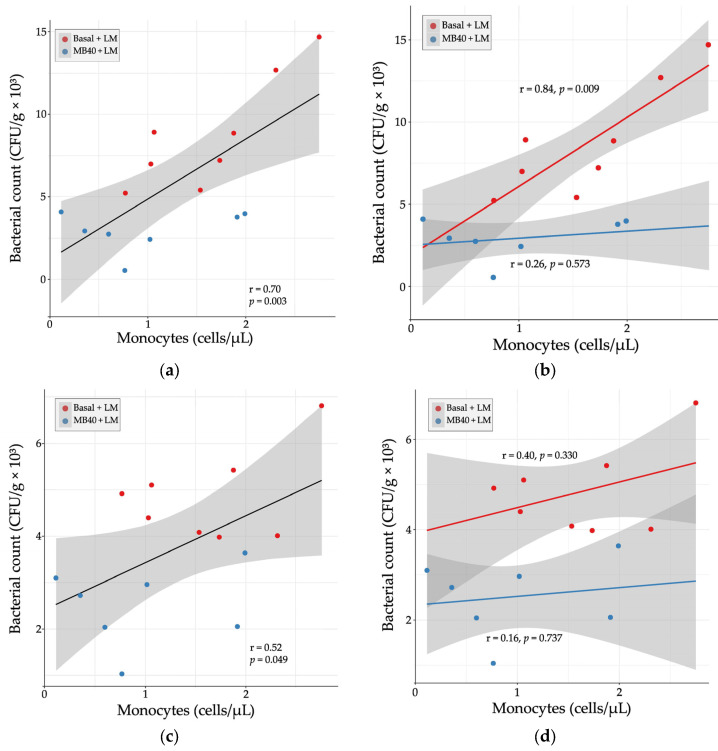
Relationship between organ bacterial counts and blood monocyte concentrations in (**a**) liver, (**b**) liver × diet, (**c**) spleen, and (**d**) spleen × diet 7 days following *Listeria* challenge in animals fed basal diet (Basal) or diet supplemented with *B. subtilis* MB40 spores (MB40). Statistical differences were determined by Pearson correlation. The areas shaded in gray indicate the 95% confidence interval.

Hematology and plasma cytokine data were also tested for correlation with organ bacterial counts in LM-challenged animals. There was a strong, positive correlation between liver bacterial count and blood monocyte concentration (r = 0.70, *p* = 0.003, Figure 4a). When separated by diet, the correlation was stronger with basal diet (r = 0.84, *p* = 0.009) and weakly correlated with MB40 supplementation (r = 0.26, *p* = 0.573, Figure 4b). In the spleen, there was a moderate, positive correlation between bacterial count and blood monocyte concentration (r = 0.52, *p* = 0.049, Figure 4c). When separated by diet, the correlations were weaker (basal diet, r = 0.40; MB40, r = 0.16) and not significant (Figure 4d).

### 3.4. Plasma Biochemistry

There was a main effect of time on several plasma biochemical parameters across this 14 d study: phosphorous (*p* < 0.001), calcium (*p* = 0.001), globulin (*p* = 0.008), alanine transaminase (*p* < 0.001), alkaline phosphatase (*p* < 0.001 sodium (*p* = 0.014), potassium (*p* < 0.001), and chloride (*p* = 0.039) (Tables S1 and S4). Despite small inter-group differences of little physiological relevance, there were significant time × LM challenge interactions for creatinine (*p* = 0.022), total protein (*p* = 0.026), albumin (*p* = 0.011), γ-glutamyltransferase (*p* = 0.001), and triglyceride (*p* = 0.001) concentrations (Table S1). A main effect of the LM challenge on increased plasma creatine kinase concentrations was observed at d15 (*p* = 0.017); however, the differences in values between experimental groups at d8, and over time to d15, suggest that LM challenge does not meaningfully impact creatine kinase levels (Table S1). Altogether, these data suggest that MB40 is safe and well-tolerated in piglets, and that the extent of LM challenge per intraperitoneal dosing of 10^8^ CFU resulted in no marked effects on liver metabolism and kidney function.

### 3.5. Intestinal Morphology

Sections of the jejunum and ileum from the small intestine and sections of the large intestine were examined for changes in epithelial morphology. There were no effects of MB40 supplementation or intraperitoneal LM challenge on jejunal VH, CD, or VH:CD ratio (Table 4). Ileal VH was comparable across both dietary treatments with and without LM challenge; however, CD was greater in LM-challenged animals irrespective of dietary treatment (*p* = 0.002, Table 4). Due to this increase in CD, VH:CD ratio was lower in LM-challenged animals (*p* = 0.009). There were no effects of dietary treatment or LM challenge on large intestinal CD (Table 4). 

## 4. Discussion

This study assessed the effect of oral *Bacillus subtilis* MB40 spore supplementation on the growth and health of piglets for 7 days before and 7 days after *Listeria monocytogenes* (LM) challenge by single intraperitoneal dose. Probiotic supplementation studies with pathogen challenge in swine have been described, but to our knowledge, this is the first study to specifically investigate *Listeria* challenge. In this new porcine listeriosis model, a single dose of LM (10^8^ CFU) was administered by intraperitoneal injection. Intraperitoneal injection has the potential to model visceral dissemination of LM between organs and particularly from the mesenteric lymph nodes to the liver and spleen, which can occur following LM invasion of the intestinal epithelium [11,77]. One week following LM dosing, animals showed increased spleen weight and higher bacterial counts in the liver and spleen, without any overt symptomology such as diarrhea or substantial weight loss. Hematological analysis confirmed that the LM dose was sufficient to affect systemic inflammation 7 days post-infection, evidenced by an increase in leukocyte, neutrophil, and monocyte concentrations in the bloodstream compared to non-challenged controls. Surprisingly, visceral LM infection did impact some intestinal morphology, as ileal crypt depth and villus height to crypt depth ratio increased and decreased, respectively. In addition to ileal villus height, all parameters of intestinal morphology in the jejunum and large intestine remained similar between LM-challenged animals and non-challenged controls, suggesting that the ileum is more sensitive to LM-related systemic inflammation. LM could also more directly compromise the intestinal epithelium by direct invasion and colonization, similar to the colonization of *Shigella flexneri* in the colonic lamina propria following intraperitoneal challenge in mice [78]. However, further studies are needed to better assess mesenteric lymph node infection, visceral spread, and intestinal colonization in our LM challenge model. Altogether, a single 10^8^ CFU intraperitoneal dose of LM in piglets recapitulated many aspects of systemic LM infection, without confounding effects of oral dosing.

We also tested the hypothesis that probiotic supplementation improves immunity in this new model of porcine listeriosis. The results show that 7 days of *Bacillus subtilis* MB40 supplementation before the LM challenge, and 7 days thereafter, reduced bacterial counts in the liver and spleen, compared to challenged animals provided basal diet without MB40. The lowering of bacterial infection in the liver and spleen with MB40 may have contributed to decreased systemic inflammation, given the lower plasma concentrations of monocytes compared to challenged animals consuming a basal diet. These data demonstrate the efficacy of administering MB40 for as little as 1 week in significantly reducing visceral LM dissemination and invasion of visceral organs. Our results align with observations from other oral pathogen challenge studies with supplementation of *Bacillus* strains in swine. In one study, weaned piglets were administered a mixture of *B. subtilis* and *B. amyloliquefaciens* for 7 days before a single oral gavage of enterotoxigenic *E. coli* F18 (ETEC F18) [60]. The probiotic group showed reduced fecal shedding of ETEC F18 7 days after infection compared to challenged piglets on a control diet, suggesting *Bacillus* strains reduced survival, growth, or intestinal attachment of ETEC F18. In a second study, weaned piglets were fed one of two *Bacillus* strains at 21 days of life onward and orally infected 7 days thereafter, for 3 continuous days, with ETEC F18 [61]. *B. subtilis* DSM 32540 or *B. pumilus* DSM 32539 supplementation led to almost 50% and 75% reductions, respectively, in mesenteric lymph node bacterial counts 21 days after infection [61]. The mechanism of *Bacillaceae* strain antibacterial action involves, in part, a suite of secreted antimicrobial compounds such as fengycins, bacteriocins, surfactins, and macrolides [32,33,79,80]. It is reasonable to project that such well-documented antimicrobial metabolites and peptides may mitigate intestinal survival, epithelial invasion, and visceral spread of pathogens between the intestine, lymph nodes, bloodstream, and organs.

Specific to *Listeria*, several antimicrobial molecules secreted by *Bacillaceae* strains have been shown to mitigate LM growth in culture [62,64,69,81,82,83,84,85,86,87,88]. In particular, a subtilin-type bacteriocin peptide from *Bacillus subtilis* JS-4 was shown by confocal laser-scanning microscopy and electron microscopy to increase LM cell membrane permeability, trigger potassium ion leakage and pore formation, and damage cell membrane integrity [89]. Following oral LM challenge in mice and rats, administration of probiotic strains from the genera *Lactobacillus*, *Lacticaseibacillus*, *Ligilactobacillus*, and *Lactococcus* was associated with increased visceral organ bacterial clearance and reduced severity of infection [90,91,92,93,94]. In one of these studies, the antilisterial effect of *Ligilactobacillus salivarius* was attributed to the production and release of a bacteriocin [91]. In the study reported here, though, LM was administered by intraperitoneal injection to model visceral LM dissemination, thus limiting this study of any direct interaction in the GI tract between LM and orally administered MB40 and any of its secreted antimicrobials. It is plausible that a stable antilisterial molecule, such as a subtilin peptide secreted by vegetative MB40 cells in the intestinal lumen, was absorbed into the circulation, leading to direct contact with and killing of LM and mitigation of visceral spread. Future studies are needed to determine whether the administration of MB40 cell-free supernatants, heat-killed postbiotic lysates, and purified bacteriocins are involved in the protective effect of MB40 in this model.

As expected, intraperitoneal LM challenge, according to our protocol, did not remarkably perturb intestinal histology. Despite the reduced reproducibility of symptoms and pathology in oral pathogen challenge experiments, as compared to intraperitoneal challenge, MB40’s role in countering LM intestinal invasion will need to be addressed in a future oral LM challenge study. The oral LM challenge will also be helpful for understanding any contributions of MB40 to gut barrier integrity during infection, which has previously been demonstrated for other *Bacillus* strains in pigs, chickens, and mice [95,96,97]. As a Gram-positive bacterium, LM does not produce endotoxin; however, it does produce other toxins, such as listeriolysin O which has been shown to disrupt the barrier integrity of human intestinal epithelial cells in cell culture [98,99,100]. It remains to be understood whether oral MB40 supplementation modulated intestinal epithelial cell function, barrier integrity, and gut immune signaling that could indirectly limit the visceral spread of LM.

Beyond gut immunity and secretion of antimicrobial molecules, MB40 could also limit the visceral spread of LM by improving systemic innate immunity, for which immunoglobulin A (IgA) is a positively correlated marker [101]. Other *Bacillaceae* strains have been shown to impact IgA levels. Dietary supplementation of *B. subtilis* DSM29784 in broiler chickens increased serum levels of IgA and IgG [97]. Lymphocytes collected from human subjects administered *Alkalihalobacillus clausii* (formerly *B. clausii*) showed a greater abundance of membrane-bound IgA and greater secretion of IgA, compared to lymphocytes from subjects administered placebo [102]. A similar probiotic preparation reduced the duration of respiratory infections in children aged 3 to 6 years of age, compared to children not receiving probiotics [103]. In a clinical study of *B. subtilis* CU1 supplementation in older adults, fecal and salivary IgA levels were increased compared to subjects receiving a placebo [104]. In addition, serum levels of the antiviral factor interferon-gamma were significantly increased by 40% after 10 days of CU1 supplementation, compared to baseline within the supplementation group, and decreased risk of respiratory infection was observed in a post hoc analysis, compared to placebo [104]. One possible explanation for this immunomodulatory activity is the direct interaction between extracellular receptors of *Bacillaceae* strains, or metabolites like short-chain fatty acids, and Toll-like receptors expressed on the surface of certain gut immune cells that lead to increased IgA production by B lymphocytes [105]. Whereas MB40 had no effects on the cytokines IL-10, TNF-α, and IL-6 in our animal study, future studies will direct attention to IgA and interferon-gamma.

Future research will also direct investigation of the effects of MB40 on the intestinal microbiome. It is of note that dietary supplementation of *B. subtilis* PB6 in sows was associated with a reduction in the fecal abundance of pathogenic species from the *Streptococcus* genus [96]. In piglets, 42 days of post-weaning supplementation with three different strains of *B. subtilis* each improved body weight, increased ileal villus height, decreased the incidence of diarrhea, and decreased fecal abundance of bacteria from the genus *Clostridium*, compared to pigs fed a control diet [106]. In a recently published, placebo-controlled clinical trial of 115 human participants, daily MB40 supplementation for 30 days remarkably promoted intestinal and nasal decolonization of *Staphylococcus aureus* [107]. Previous work in mice showed that intestinal decolonization of *S. aureus* is dependent on the production and secretion of fengycin from *B. subtilis* [79]. Given this pathogenic species-specific effect, future microbiome analysis will comprise metagenomic sequencing to more comprehensively understand the strain-specific effects of MB40 on the mammalian gut microbiome.

## 5. Conclusions

The data presented in this study indicate that the inclusion of the probiotic *Bacillus subtilis* MB40 spores in the diet of nursery piglets helps improve innate immunity and disease resistance in an animal model of listeriosis. In this new porcine model, a single intraperitoneal dose of *Listeria monocytogenes* led to the dissemination of bacteria to the liver and spleen, increased spleen weight, and higher concentrations of circulating leukocytes, neutrophils, and monocytes, compared to non-challenged controls. Remarkably, animals supplemented with *B. subtilis* MB40 showed reduced bacterial counts in the liver and spleen and lower plasma monocyte concentrations following this *Listeria* challenge. Because the pathogen challenge was via intraperitoneal injection, it is likely that orally administered *B. subtilis* MB40 spores germinate in the intestine and secrete an antilisterial molecule that is absorbed and mitigates visceral dissemination of *Listeria*. Further studies are needed to elucidate MB40’s mechanism of action and investigate MB40’s utility in helping manage foodborne illness and promoting gut microbiota balance in humans. Altogether, *B. subtilis* MB40 is a promising probiotic in humans and animals for preventing, mitigating, and alleviating symptoms of listeriosis.

## Figures and Tables

**Table 1:** Effects of *B. subtilis* MB40 supplementation and *Listeria* challenge on growth parameters ^1^.

	Condition				*p* Value		
Parameter (Unit)	Basal	Basal + LM	MB40	MB40 + LM	Diet	Challenge	D × C
Body weight (kg) *							
d1	7.46 ± 1.67	6.96 ± 1.97	6.92 ± 1.64	7.16 ± 1.78	0.785	0.840	0.561
d8	9.07 ± 2.01	8.60 ± 2.49	8.74 ± 2.03	8.96 ± 2.90	0.985	0.881	0.692
d15	12.83 ± 2.92	12.04 ± 3.37	11.94 ± 2.98	11.98 ± 3.30	0.672	0.739	0.717
Body weight gain (kg) *							
d1–d8	1.61 ± 0.67	1.64 ± 1.10	1.83 ± 0.70	1.80 ± 1.21	0.580	0.999	0.929
d8–d15	3.76 ± 1.87	3.44 ± 1.46	3.20 ± 1.11	3.02 ± 0.64	0.311	0.608	0.885
d1–d15	5.37 ± 2.24	5.08 ± 1.50	5.03 ± 1.51	4.82 ± 1.66	0.628	0.693	0.950
ADG (kg) *							
d1–d8	0.18 ± 0.07	0.20 ± 0.11	0.19 ± 0.12	0.17 ± 0.09	0.727	0.963	0.647
d8–d15	0.18 ± 0.07	0.18 ± 0.12	0.20 ± 0.08	0.20 ± 0.13	0.579	0.995	0.929
d1–d15	0.42 ± 0.21	0.38 ± 0.16	0.36 ± 0.12	0.34 ± 0.07	0.309	0.608	0.886
ADFI (kg) *							
d1–d8	0.50 ± 0.14	0.54 ± 0.17	0.45 ± 0.13	0.47 ± 0.18	0.313	0.253	0.919
d8–d15	0.81 ± 0.16	0.76 ± 0.17	0.73 ± 0.17	0.73 ± 0.19	0.953	0.898	0.871
Total feed intake (kg)							
d1–d15	9.95 ± 1.83	9.85 ± 2.44	9.00 ± 2.12	9.14 ± 2.73	0.316	0.978	0.879
FCR (kg/kg)							
d1–d8	2.44 ± 0.96	2.57 ± 1.29	1.89 ± 0.60	1.97 ± 0.63	0.096	0.765	0.932
d8–d15	1.69 ± 0.53	1.97 ± 0.69	1.97 ± 0.63	1.93 ± 0.31	0.550	0.539	0.437
d1–d15 ^‡^	1.76 ± 0.31 ^A^	2.00 ± 0.36 ^B^	1.72 ± 0.12 ^A^	1.93 ± 0.21 ^B^	0.593	***0.031***	0.902

^1^ Data are means ± standard deviation (*n* = 7–8 per group); * main effect of time; ^‡^ main effect of LM challenge; significant differences between groups (*p* < 0.05, ANOVA) are denoted by unshared letters (challenge factor: A, B); significant *p* values are italicized and bold (*p* values for three-way repeated measures ANOVA are shown in Table S1). Abbreviations: ADG, average daily weight gain; ADFI, average daily feed intake; Basal, basal diet; d, day; D × C, diet × challenge interaction; FCR, feed conversion ratio; LM, *Listeria monocytogenes* challenge at d8; MB40, MB40-supplemented diet.

**Table 2:** Effects of *B. subtilis* MB40 supplementation and *Listeria* challenge on organ weights ^1^.

	Condition				*p* Value		
Parameter (Unit)	Basal	Basal + LM	MB40	MB40 + LM	Diet	Challenge	D × C
Small intestine weight (g) ^†^	535 ± 111 ^a^	620 ± 81 ^a^	505 ± 70 ^b^	510 ± 83 ^b^	***0.036***	0.166	0.219
Liver weight (g)	401 ± 75	405 ± 107	410 ± 113	405 ± 68	0.894	0.999	0.901
Kidney weight (g)	36 ± 9	40 ± 10	35 ± 14	38 ± 6	0.635	0.305	0.851
Spleen weight (g)	27 ± 10	33 ± 10	26 ± 14	31 ± 10	0.664	0.207	0.934
Normalized small intestine weight (% bw)	6.3 ± 0.61	6.5 ± 0.98	5.9 ± 1.71	6.1 ± 0.97	0.294	0.687	0.896
Normalized liver weight (% bw)	3.3 ± 0.46	3.2 ± 0.53	3.3 ± 0.88	3.4 ± 0.76	0.651	0.976	0.726
Normalized kidney weight (% bw)	0.29 ± 0.02	0.33 ± 0.03	0.27 ± 0.08	0.32 ± 0.1	0.510	0.052	0.801
Normalized spleen weight (% bw) ^‡^	0.22 ± 0.07 ^A^	0.26 ± 0.04 ^B^	0.20 ± 0.09 ^A^	0.27 ± 0.03 ^B^	0.795	***0.017***	0.585

^1^ Data are means ± standard deviation (*n* = 8 per group); ^†^ main effect of diet; ^‡^ main effect of LM challenge; significant differences between groups (*p* < 0.05, ANOVA) are denoted by unshared letters (diet factor: a, b; challenge factor: A, B); significant *p* values are italicized and bold. Abbreviations: Basal, basal diet; bw, body weight; D × C, diet × challenge interaction; LM, *Listeria monocytogenes* challenge at d8; MB40, MB40-supplemented diet.

**Table 4:** Effect of MB40 supplementation and *Listeria* challenge on intestinal morphology ^1^.

	Condition				*p* Value		
Parameter (Unit)	Basal	Basal + LM	MB40	MB40 + LM	Diet	Challenge	D × C
Jejunum							
Villus height (µM)	490 ± 67	473 ± 51	437 ± 98	471 ± 66	0.298	0.746	0.334
Crypt depth (µM)	210 ± 50	227 ± 54	195 ± 17	202 ± 17	0.156	0.402	0.721
VH:CD ratio	2.46 ± 0.77	2.19 ± 0.59	2.25 ± 0.55	2.44 ± 0.26	0.919	0.845	0.271
Ileum							
Villus height (µM)	454 ± 71	436 ± 38	432 ± 108	400 ± 114	0.360	0.417	0.822
Crypt depth (µM) ^‡^	189 ± 25 ^A^	244 ± 48 ^B^	185 ± 30 ^A^	231 ± 57 ^B^	0.548	***0.002***	0.773
VH:CD ratio ^‡^	2.43 ± 0.35 ^A^	1.86 ± 0.48 ^B^	2.35 ± 0.48 ^A^	1.85 ± 0.74 ^B^	0.818	***0.009***	0.870
Large intestine							
Crypt depth (µM)	324 ± 59	324 ± 41	293 ± 74	298 ± 51	0.178	0.895	0.885

^1^ Data are means ± standard deviation (*n* = 7–8 per group); ^‡^ main effect of LM challenge; significant differences between groups (*p* < 0.05, ANOVA) are denoted by unshared letters (challenge factor: A, B); significant *p* values are italicized and bold. Abbreviations: Basal, basal diet; D × C, diet × challenge interaction; LM, *Listeria monocytogenes* challenge; MB40, MB40-supplemented diet; VH:CD ratio, villus height to crypt depth ratio.

## Data Availability

Data not presented within the article or Supplementary Materials are available upon reasonable request from the corresponding authors.

## Supplementary Materials

The following supporting information can be downloaded at: https://www.mdpi.com/article/10.3390/microorganisms11082110/s1, Table S1: Effects of *B. subtilis* MB40 supplementation and *Listeria* challenge on plasma biochemistry; Table S2: *p* values for effects of *B. subtilis* MB40 supplementation and *Listeria* challenge on growth parameters; Table S3: *p* values for the effects of *B. subtilis* MB40 supplementation and *Listeria* challenge on hematology and plasma cytokines; Table S4: *p* values for the effects of *B. subtilis* MB40 supplementation and *Listeria* challenge on plasma biochemistry.
