# Supplementary material for: The Probiotic Bacillus subtilis MB40 Improves Immunity in a Porcine Model of Listeriosis
Source: Microorganisms. 2023 Aug 18;11(8):2110. doi: 10.3390/microorganisms11082110 (PMC10458092; doi:10.3390/microorganisms11082110)
Supplement: Supplementary file 1 [file microorganisms-11-02110-s001.zip › microorganisms-2465341-supplementary.pdf]

microorganisms

Supplementary Material

The Probiotic *Bacillus subtilis* MB40 Improves Immunity in a Porcine Model of Listeriosis

Sean M. Garvey [1], Nima K. Emami [2], Justin L. Guice [1], Nammalwar Sriranganathan [3], Christopher Penet [1], Robert P. Rhoads [2], Jessica L. Spears [4,*], Rami A. Dalloul [5], and Samer W. El-Kadi [2,*]

[1] Department of Research and Development, BIO-CAT, Inc., Troy, VA 22974, USA
[2] School of Animal Sciences, Virginia Tech, Blacksburg, VA 24061, USA
[3] Department of Biomedical Sciences and Pathobiology, Virginia Tech, Blacksburg, VA 24061, USA
[4] Department of Research and Development, BIO-CAT Microbials, LLC, Shakopee, MN 55379, USA
[5] Department of Poultry Science, University of Georgia, Athens, GA 30602, USA
* Correspondence: jspears@bio-cat.com (J.L.S.); elkadi@vt.edu (S.W.E.-K.)

Table S1. Effects of MB40 supplementation and *Listeria* challenge on plasma biochemistry. [1]

	Condition				*p* Value		
**Parameter (Unit)**	**Basal**	**Basal + LM**	**MB40**	**MB40 + LM**	**Diet**	**Challenge**	**D × C**
Glucose (mg/dL)							
d1	117.3 ± 15.9	113.5 ± 9.3	123.6 ± 9.7	119.5 ± 11.0	0.159	0.366	0.970
d8 †	115.1 ± 12.7 [a]	117.9 ± 6.2 [a]	123.3 ± 12.0 [b]	124.4 ± 5.1 [b]	***0.046***	0.580	0.824
d15	125.6 ± 24.0	118.1 ± 11.7	120.9 ± 9.3	133.3 ± 19.9	0.403	0.693	0.115
Urea nitrogen (mg/dL) ‡							
d1 ‡	14.8 ± 4.06 [A]	12.1 ± 1.81 [B]	14.5 ± 2.07 [A]	12.9 ± 3.04 [B]	0.808	***0.047***	0.628
d8	14.6 ± 2.45	12.8 ± 2.12	13.5 ± 1.77	13.1 ± 2.23	0.627	0.151	0.334
d15	15.0 ± 2.33	13.3 ± 2.43	14.6 ± 2.77	14.1 ± 2.47	0.780	0.215	0.487
Creatinine (mg/dL) *,⁂							
d1	0.67 ± 0.14	0.62 ± 0.10	0.75 ± 0.09	0.63 ± 0.12	0.256	0.057	0.470
d8	0.56 ± 0.05	0.58 ± 0.11	0.62 ± 0.04	0.64 ± 0.10	0.067	0.531	0.948
d15	0.60 ± 0.07	0.60 ± 0.09	0.63 ± 0.04	0.61 ± 0.09	0.497	0.690	0.690
Phosphorous (mg/dL) *							
d1	7.7 ± 1.00	7.9 ± 1.27	7.9 ± 1.23	7.7 ± 0.62	0.947	0.869	0.621
d8	8.8 ± 0.63	8.8 ± 0.91	8.5 ± 0.63	9.1 ± 0.82	0.908	0.346	0.261
d15	8.9 ± 0.87	9.0 ± 0.98	9.0 ± 0.44	9.2 ± 0.61	0.578	0.488	0.853
Calcium (mg/dL) *							
d1	11.1 ± 1.08	11.0 ± 1.00	10.9 ± 1.15	10.7 ± 0.88	0.577	0.722	0.879
d8	10.1 ± 0.45	10.4 ± 0.44	10.3 ± 0.54	10.3 ± 0.50	0.640	0.496	0.542
d15	10.5 ± 0.36	10.6 ± 0.29	10.6 ± 0.71	10.6 ± 0.35	0.939	0.594	0.703
Total protein (g/dL)							
d1	4.7 ± 0.63	4.5 ± 0.44	4.8 ± 0.60	4.5 ± 0.41	0.576	0.228	0.766
d8	4.1 ± 0.33	4.4 ± 0.30	4.3 ± 0.38	4.2 ± 0.31	0.600	0.403	0.122
d15	4.3 ± 0.20	4.5 ± 0.29	4.4 ± 0.46	4.4 ± 0.36	0.877	0.643	0.283
Albumin (g/dL) *,⁂							
d1	2.7 ± 0.43	2.7 ± 0.33	2.9 ± 0.39	2.6 ± 0.26	0.660	0.266	0.526
d8	2.4 ± 0.46	2.6 ± 0.34	2.5 ± 0.28	2.5 ± 0.40	0.889	0.431	0.609
d15	2.4 ± 0.33	2.6 ± 0.33	2.5 ± 0.29	2.5 ± 0.37	0.712	0.563	0.373
Globulin (g/dL) *							
d1	1.9 ± 0.33	1.8 ± 0.22	2.0 ± 0.41	1.9 ± 0.17	0.598	0.382	0.860
d8	1.7 ± 0.22	1.8 ± 0.21	1.9 ± 0.25	1.7 ± 0.19	0.521	0.999	0.155
d15	1.9 ± 0.15	1.9 ± 0.20	2.0 ± 0.26	1.9 ± 0.17	0.773	0.890	0.660

Alanine transaminase (U/L) *							
d1 ‡,⁑	19.9 ± 3.76 [A]	19.5 ± 3.66 [B]	25.3 ± 5.28 [A]	17.8 ± 4.30 [B]	0.243	***0.015***	***0.027***
d8	24.9 ± 5.57	24.0 ± 4.81	25.3 ± 5.55	24.3 ± 5.28	0.869	0.622	0.974
d15	31.1 ± 4.82	30.8 ± 6.76	28.4 ± 4.93	29.4 ± 6.40	0.352	0.884	0.749
Alkaline phosphatase (U/L) *							
d1	362 ± 73.4	395 ± 77.8	390 ± 81.7	340 ± 78.7	0.626	0.762	0.145
d8	314 ± 69.2	348 ± 72.3	314 ± 70.4	312 ± 44.1	0.434	0.479	0.447
d15	344 ± 64.4	340 ± 103.2	332 ± 63.2	293 ± 44.3	0.254	0.407	0.487
Gamma-glutamyl-transferase (U/L) ⁂							
d1	28.6 ± 8.77	34.6 ± 11.69	33.4 ± 8.12	29.8 ± 7.48	0.985	0.717	0.148
d8 ⁑	27.0 ± 3.70	35.0 ± 12.60	32.5 ± 5.88	25.9 ± 3.76	0.867	0.050	***0.033***
d15	30.7 ± 1.27	31.0 ± 1.30	35.6 ± 1.28	29.8 ± 1.20	0.507	0.324	0.265
Creatine kinase (mg/dL)							
d1 ⁑	281 ± 93.3	447 ± 330.1	468 ± 188.5	246 ± 116.0	0.931	0.716	***0.020***
d8	641 ± 489.4	885 ± 905.4	461 ± 391.3	361 ± 207.5	0.080	0.909	0.404
d15 ‡	469 ± 241.6 [A]	694 ± 457.2 [B]	591 ± 434.3 [A]	1278 ± 687.1 [B]	0.058	***0.017***	0.206
Cholesterol (mg/dL)							
d1	82.4 ± 20.11	76.3 ± 8.61	79.8 ± 27.26	73.8 ± 13.93	0.703	0.370	0.993
d8	80.6 ± 17.10	80.3 ± 12.37	77.0 ± 10.65	80.1 ± 7.26	0.671	0.756	0.692
d15	87.4 ± 13.32	86.3 ± 13.55	82.9 ± 14.16	82.6 ± 11.59	0.391	0.884	0.926
Triglycerides (mg/dL) ‡,⁂							
d1 ‡	55.4 ± 12.66 [A]	39.4 ± 12.45 [B]	61.8 ± 21.31 [A]	36.4 ± 9.58 [B]	0.747	***< 0.001***	0.374
d8	45.0 ± 10.64	40.5 ± 8.43	36.3 ± 10.95	44.5 ± 12.07	0.542	0.630	0.107
d15	48.0 ± 17.15	45.4 ± 10.03	46.5 ± 14.64	45.8 ± 14.43	0.912	0.741	0.854
Sodium (mEq/L) *							
d1	143 ± 2.3	143 ± 2.7	143 ± 3.5	144 ± 2.2	0.964	0.782	0.741
d8	141 ± 1.3	141 ± 2.9	142 ± 3.0	142 ± 1.6	0.179	0.880	0.880
d15	142 ± 2.3	143 ± 2.5	141 ± 2.6	142 ± 2.2	0.533	0.186	0.926
Potassium (mEq/L) *							
d1	5.1 ± 0.60	5.0 ± 0.69	4.8 ± 0.77	4.5 ± 0.29	0.086	0.349	0.441
d8	5.6 ± 0.88	5.2 ± 0.38	5.4 ± 0.89	5.4 ± 0.69	0.999	0.372	0.397
d15	5.5 ± 0.68	5.3 ± 0.36	5.7 ± 0.72	5.4 ± 0.96	0.448	0.253	0.980
Chloride (mEq/L) *							
d1	104 ± 4.4	102 ± 3.0	104 ± 4.6	103 ± 1.9	0.666	0.317	0.666
d8	103 ± 2.4	102 ± 1.6	102 ± 2.4	101 ± 1.6	0.305	0.492	0.863
d15	102 ± 2.2	101 ± 1.4	102 ± 3.0	102 ± 1.6	0.675	0.288	0.471
Carbon dioxide (mEq/L)							
d1	26.6 ± 3.54	26.8 ± 2.87	24.3 ± 3.54	26.0 ± 0.76	0.140	0.370	0.436
d8	25.0 ± 4.66	24.8 ± 3.20	26.3 ± 3.28	25.4 ± 3.29	0.475	0.667	0.811
d15	24.3 ± 4.20	28.0 ± 2.78	26.5 ± 3.85	26.1 ± 2.80	0.880	0.179	0.104
Anion gap (mEq/L)							
d1	19.9 ± 2.97	19.4 ± 2.50	22.2 ± 3.91	18.8 ± 2.24	0.426	0.074	0.190
d8	19.0 ± 3.02	19.2 ± 3.01	19.4 ± 2.84	20.6 ± 3.39	0.395	0.525	0.625
d15	20.7 ± 3.64	19.3 ± 4.23	19.1 ± 1.44	19.7 ± 3.12	0.646	0.732	0.389

[1] Data are means ± standard deviation (n = 6–8 per group); † main effect of diet; ‡ main effect of LM challenge; * main effect of time; ⁑ diet × LM challenge interaction; ⁂ time × LM challenge interaction; significant differences between groups ($p < 0.05$, ANOVA) are denoted by unshared letters (diet factor: a, b; challenge factor: A, B); significant p values are italicized and bolded. Abbreviations: Basal, basal diet; d, day; D × C, diet × challenge interaction; LM, *Listeria monocytogenes* challenge at d8; MB40, MB40-supplemented diet.

Table S2. *p* values for the effects of *B. subtilis* MB40 supplementation and *Listeria* challenge on growth parameters. [1]

Parameter (Unit)	Diet × Challenge × Time	Diet × Challenge	Diet × Time	Challenge × Time	Diet	Challenge	Time
Body weight (kg)	0.990	0.661	0.603	0.845	0.803	0.804	***< 0.001***
Body weight gain(kg)	0.976	0.950	0.320	0.825	0.628	0.693	***< 0.001***
ADG (kg)	0.856	0.951	0.220	0.650	0.627	0.696	***< 0.001***
ADFI (kg)	0.382	0.898	0.958	0.188	0.313	0.951	***< 0.001***
FCR (kg/kg)	0.737	0.629	0.077	0.949	0.246	0.561	0.094

[1] Significant differences for factors ($p < 0.05$, three-way repeated measures ANOVA) are italicized and bolded (n = 7–8 per group). Abbreviations: ADG, average daily weight gain; ADFI, average daily feed intake; FCR, feed conversion ratio.

Table S3. *p* values for the effects of *B. subtilis* MB40 supplementation and *Listeria* challenge on hematology and plasma cytokines. [1]

Parameter (Unit)	Diet × Challenge × Time	Diet × Challenge	Diet × Time	Challenge × Time	Diet	Challenge	Time
Erythrocytes (cells/μL)	0.564	0.389	0.790	0.431	0.945	0.988	**0.039**
Hemoglobin (g/dL)	0.502	0.106	0.516	0.730	0.999	0.275	***< 0.001***
Hematocrit (%)	0.943	0.110	0.458	0.470	0.903	0.347	***< 0.001***
Packed cell volume (%)	0.831	0.303	0.387	0.085	0.504	0.923	**0.001**
Leukocytes (cells/μL)	0.786	0.276	0.513	0.137	0.247	0.027	0.075
Segmented neutrophils (cells/μL)	0.913	0.877	0.265	0.440	0.555	***0.004***	***0.020***
Lymphocytes (cells/μL)	0.713	0.081	0.920	0.693	0.248	0.632	0.087
Eosinophils (cells/μL)	0.156	0.522	***0.032***	0.105	0.668	0.499	0.456
Monocytes (cells/μL)	0.298	0.806	***0.045***	0.196	0.468	***0.009***	0.449
Interleukin-10 (pg/mL)	0.607	0.218	0.841	0.605	0.739	0.584	***0.018***
Tumor necrosis factor-α (pg/mL)	0.886	0.724	0.154	0.371	0.290	0.381	***0.023***
Interleukin-6 (pg/mL)	0.683	0.387	0.638	0.538	0.797	0.444	***< 0.001***

[1] Significant differences for factors ($p < 0.05$, three-way repeated measures ANOVA) are italicized and bolded (n = 6–8 per group).

Table S4. *p* values for the effects of *B. subtilis* MB40 supplementation and *Listeria* challenge on plasma biochemistry. [1]

Parameter (Unit)	Diet × Challenge × Time	Diet × Challenge	Diet × Time	Challenge × Time	Diet	Challenge	Time
Glucose (mg/dL)	0.120	0.412	0.955	0.488	0.073	0.984	0.136
Urea nitrogen (mg/dL)	0.040	1.515	0.337	0.862	***0.007***	8.246	1.791
Creatinine (mg/dL)	0.670	0.612	0.564	***0.022***	0.124	0.338	***0.003***
Phosphorous (mg/dL)	0.540	0.745	0.951	0.751	0.691	0.464	***< 0.001***
Calcium (mg/dL)	0.981	0.703	0.553	0.614	0.849	0.899	***0.001***
Total protein (g/dL)	0.595	0.326	0.617	***0.026***	0.692	0.843	***< 0.001***
Albumin (g/dL)	0.906	0.466	0.506	***0.011***	0.929	0.929	***< 0.001***
Globulin (g/dL)	0.375	0.522	0.939	0.554	0.522	0.604	***0.008***
Alanine transaminase (U/L)	0.243	0.528	0.848	0.272	0.511	0.336	***< 0.001***
Alkaline phosphatase (U/L)	0.550	0.233	0.798	0.302	0.338	0.834	***< 0.001***
Gamma-glutamyltransferase (U/L)	0.530	0.086	0.560	***0.001***	0.875	0.659	0.083
Creatine kinase (mg/dL)	0.370	0.578	0.160	0.182	0.833	0.086	0.058
Cholesterol (mg/dL)	0.964	0.843	0.943	0.507	0.457	0.637	0.116

Triglycerides (mg/dL)	0.217	0.810	0.824	***0.001***	0.922	***0.039***	0.103
Sodium (mEq/L)	0.534	0.737	0.653	0.909	0.507	0.821	***0.014***
Potassium (mEq/L)	0.478	0.909	0.175	0.957	0.676	0.146	***< 0.001***
Chloride (mEq/L)	0.846	0.834	0.399	0.743	0.741	0.134	***0.039***
Carbon dioxide (mEq/L)	0.207	0.488	0.288	0.369	0.845	0.361	0.548
Anion gap (mEq/L)	0.117	0.957	0.402	0.100	0.626	0.521	0.649

[1] Significant differences for factors ($p < 0.05$, three-way repeated measures ANOVA) are italicized and bolded (n = 6–8 per group).
